# Supplementary material for: The in vivo genetic program of murine primordial lung epithelial progenitors
Source: Nat Commun. 2020 Jan 31;11:635. doi: 10.1038/s41467-020-14348-3 (PMC6994558; doi:10.1038/s41467-020-14348-3)
Supplement: Supplementary file 1 — Description of Additional Supplementary Files [file 41467_2020_14348_MOESM1_ESM.pdf]

Title: Supplementary Data 1.
Description: Pairwise comparisons of gene expression for conditions depicted in Figure 2B

Title: Supplementary Data 2.
Description: The top 500 most variable genes used in the Principal Component Analysis of Figure 5I

Title: Supplementary Data 3.
Description: Linear Algebra Projection (LAP) scores
