# Supplementary material for: The in vivo genetic program of murine primordial lung epithelial progenitors
Source: Nat Commun. 2020 Jan 31;11:635. doi: 10.1038/s41467-020-14348-3 (PMC6994558; doi:10.1038/s41467-020-14348-3)
Supplement: Supplementary file 3 — Reporting Summary [file 41467_2020_14348_MOESM3_ESM.pdf]

nature research

Corresponding author(s): Laertis Ikonomou
Darrell N. Kotton

Last updated by author(s): Dec 18, 2019

Reporting Summary

Nature Research wishes to improve the reproducibility of the work that we publish. This form provides structure for consistency and transparency in reporting. For further information on Nature Research policies, see Authors & Referees and the Editorial Policy Checklist .

Statistics

For all statistical analyses, confirm that the following items are present in the figure legend, table legend, main text, or Methods section.

n/a	Confirmed	
☐	☒	The exact sample size (*n*) for each experimental group/condition, given as a discrete number and unit of measurement
☒	☐	A statement on whether measurements were taken from distinct samples or whether the same sample was measured repeatedly
☐	☒	The statistical test(s) used AND whether they are one- or two-sided<br>*Only common tests should be described solely by name; describe more complex techniques in the Methods section.*
☐	☒	A description of all covariates tested
☐	☒	A description of any assumptions or corrections, such as tests of normality and adjustment for multiple comparisons
☐	☒	A full description of the statistical parameters including central tendency (e.g. means) or other basic estimates (e.g. regression coefficient) AND variation (e.g. standard deviation) or associated estimates of uncertainty (e.g. confidence intervals)
☒	☐	For null hypothesis testing, the test statistic (e.g. *F*, *t*, *r*) with confidence intervals, effect sizes, degrees of freedom and *P* value noted<br>*Give P values as exact values whenever suitable.*
☒	☐	For Bayesian analysis, information on the choice of priors and Markov chain Monte Carlo settings
☒	☐	For hierarchical and complex designs, identification of the appropriate level for tests and full reporting of outcomes
☒	☐	Estimates of effect sizes (e.g. Cohen's *d*, Pearson's *r*), indicating how they were calculated

Our web collection on statistics for biologists contains articles on many of the points above.

Software and code

Policy information about availability of computer code

Data collection	Data collection is described in the Methods section.
Data analysis	Data analysis is described in the Methods section.

For manuscripts utilizing custom algorithms or software that are central to the research but not yet described in published literature, software must be made available to editors/reviewers. We strongly encourage code deposition in a community repository (e.g. GitHub). See the Nature Research guidelines for submitting code & software for further information.

Data

Policy information about availability of data

All manuscripts must include a data availability statement. This statement should provide the following information, where applicable:

- Accession codes, unique identifiers, or web links for publicly available datasets
- A list of figures that have associated raw data
- A description of any restrictions on data availability

All custom LAP scripts have been made available at [https://github.com/Emergent-Behaviors-in-Biology/lung-primordium]. Additional modified scripts can be accessed upon request. All sequencing data that support the findings of this study have been deposited in the National Center for Biotechnology Information Gene Expression Omnibus (GEO) and are accessible through the following GEO Series accession numbers:
a) In vivo bulk RNA-Seq: E8.25 Foregut Endoderm and Ectoderm, E9.0 Nkx2-1+ Lung and Brain, E9.0 Nkx2-1- Lung and Brain, E13.5 Nkx2-1+ Thyroid, E13.5 Nkx2-1- Thyroid; GEO Series accession number GSE138903
b) In vitro bulk RNA-Seq: 2D-Nkx2-1+EPCAM+, 3D-Nkx2-1+EPCAM+, 2D-Nkx2-1-EPCAM+; GEO Series accession number GSE138676
c) In vivo E9.0 single-cell RNA-Seq; GEO Series accession number GSE138904
d) E13.5 lung Nkx2-1GFP+ single-cell data; GEO Series accession number GSE139186
The E15.5 and E17.5 lung Nkx2-1GFP+ single -cell data had been previously deposited and are accessible through GEO Series accession number GSE113320: [https://www.ncbi.nlm.nih.gov/geo/query/acc.cgi?acc=GSE113320].

The microarray data containing the 2D-Nkx2-1+ condition had been previously deposited and are accessible through GEO series accession number GSE92916: [https://www.ncbi.nlm.nih.gov/geo/query/acc.cgi?acc=GSE92916].
The bulk RNA-Seq for the thyroid directed differentiation (D1, D7, D14 conditions) had been previously deposited and are accessible thorough GEO series accession number GSE92572: [https://www.ncbi.nlm.nih.gov/geo/query/acc.cgi?acc=GSE92572].
All other relevant data are available from the corresponding author upon reasonable request.

Field-specific reporting

Please select the one below that is the best fit for your research. If you are not sure, read the appropriate sections before making your selection.

☒ Life sciences ☐ Behavioural & social sciences ☐ Ecological, evolutionary & environmental sciences

For a reference copy of the document with all sections, see nature.com/documents/nr-reporting-summary-flat.pdf

Life sciences study design

All studies must disclose on these points even when the disclosure is negative.

Sample size	For bulk RNA-Seq, we used three biological replicates for each condition (cells sorted from three independent litter pools). For in vitro directed differentiation experiments, we used data from three independent experiments.
Data exclusions	No data were excluded from the analysis.
Replication	All attempts at replication were successful.
Randomization	For lineage tracing studies, animals were randomly assigned to tamoxifen-treated or control (no treatment) groups.
Blinding	No blinding was attempted.

Reporting for specific materials, systems and methods

We require information from authors about some types of materials, experimental systems and methods used in many studies. Here, indicate whether each material, system or method listed is relevant to your study. If you are not sure if a list item applies to your research, read the appropriate section before selecting a response.

Materials & experimental systems

n/a	Involved in the study	
☐	☒	Antibodies
☐	☒	Eukaryotic cell lines
☒	☐	Palaeontology
☐	☒	Animals and other organisms
☒	☐	Human research participants
☒	☐	Clinical data

Methods

n/a	Involved in the study	
☒	☐	ChIP-seq
☐	☒	Flow cytometry
☒	☐	MRI-based neuroimaging

Antibodies

Antibodies used	For immunohistochemistry, the following antibodies were used: goat anti-SCGB1A1 (1:200, Santa Cruz, Cat No: sc-9772), goat anti-SCGB1A1 (1:200, Sigma-Aldrich, Cat No: 07-623), rabbit anti-pro-SPC (1:200 or 1:1000, Seven Hill Bioreagents, Cat No: WRAB-9337), hamster anti-mouse PDPN1 monoclonal antibody (1:200, Developmental Studies Hybridoma Bank, 8.1.1), rabbit anti-acetylated α-tubulin (1:800, Cell Signaling Technology 5335), rabbit anti-P63-alpha (1:100, Cell Signaling Technology 13109), rabbit anti-NKX2-1 (1:200, abcam, Cat No: ab76013), chicken anti-GFP (1:50, Invitrogen A10262), anti-E-Cadherin (phospho S838 + S840) (1:200, abcam, Cat No: ab76319), APC-Cy7-conjugated anti-mouse rat EPCAM antibody (Biolegend CD326, Cat No: 118217).<br>For immunocytochemistry, the following antibodies were used: rat anti-CDH1 antibody (Sigma-Aldrich, Cat No: U3254), rabbit anti-NKX2-1 (1:200, abcam, Cat No: ab76013).<br>For flow cytometry the following antibodies were used: APC/Cy7 anti-mouse CD326 (1:80, Biolegend, Cat No: 118218, clone G8.8), ENDM1 primary antibody (1:100, DMBC2-8-610) (Gadue et al., Stem Cells, 2009).
Validation	All in vivo antibody stains correlated well with the predicted anatomical and cell type -specific subcellular location. All cell fractions sorted by flow cytometry using specific antibodies, had highly enriched gene expression for the corresponding genes.

Eukaryotic cell lines

Policy information about cell lines

Cell line source(s)	Nkx2-1mCherry mouse embryonic stem cell (ESC) line (Bilodeau et al., Stem Cell Reports, 2014).
Authentication	The Nkx2-1mCherry mouse embryonic stem cell (ESC) line was found to be euploid by G-banding. The mCherry reporter recapitulated endogenous Nkx2-1 locus expression in lung/thyroid directed differentiation (Serra et al., Development, 2017; Kurmann et al., Cell Stem Cell, 2015; current work).
Mycoplasma contamination	All cell lines used in this work have been tested and found free of mycoplasma contamination.
Commonly misidentified lines (See ICLAC register)	We have not used any commonly misidentified cell lines.

Animals and other organisms

Policy information about studies involving animals; ARRIVE guidelines recommended for reporting animal research

Laboratory animals	The following mouse strains were used: Nkx2-1-GFP, Nkx2-1CreERT2 (Nkx2-1tm1.1(cre/ERT2)Zjh/), R26RnT/nG (B6;129S6-Gt (ROSA)26Sortm1(CAG-tdTomato*,-EGFP*)Ees/), Nkx2.1-Cre (C57BL/6J-Tg(Nkx2-1-cre)2Sand/J), and R26R (B6.129S4-Gt(ROSA) 26Sortm1Sor/J). Both male and female animals, less than 1-year old, were used.
Wild animals	N/A
Field-collected samples	N/A
Ethics oversight	All mouse studies involving mice carrying GFP, CreERT2, Cre and nT/nG transgenes were approved by the Institutional Animal Care and Use Committee of Boston University School of Medicine. All mouse studies involving mouse foregut explants were approved by the Institutional Animal Care and Use Committee of Cincinnati Children's Hospital.

Note that full information on the approval of the study protocol must also be provided in the manuscript.

Flow Cytometry

Plots

Confirm that:

- [x] The axis labels state the marker and fluorochrome used (e.g. CD4-FITC).
- [x] The axis scales are clearly visible. Include numbers along axes only for bottom left plot of group (a 'group' is an analysis of identical markers).
- [x] All plots are contour plots with outliers or pseudocolor plots.
- [x] A numerical value for number of cells or percentage (with statistics) is provided.

Methodology

Sample preparation	For E8.25 embryos (5-6 somite stage), we used a published protocol to sort foregut endoderm and ectoderm (Gadue et al., Stem Cells, 2009). Briefly, Nkx2-1-GFP embryos were incubated in trypsin for up to 3 minutes and the cell monodispersion was incubated with the appropriate antibodies and viability dyes (see Methods).<br>For E9.0 and E13.5 embryos, the three Nkx2-1GFP-expressing domains (forebrain, thyroid, and lung) were dissected out using Tungsten needles with the help of an Olympus stereo fluorescence imaging microscope and kept in HBSS with 10% FBS. Tissues were digested using Collagenase A (0.1%, Roche 103578) and Dispase II (2.4 U ml-1, Roche 295825) supplemented with 2.5 mM CaCl2 for 60 min at 37ºC with periodic trituration. Monodispersed cells were resuspended in HBSS+ buffer, filtered through 30 µm FACS strainers (Miltenyi Biotech, Cat No: 130-041-407) and stained with viability dyes (see Methods).<br>Lungs from adult Nkx2-1GFP mice were extracted, minced and digested using Collagenase A (0.1%, Roche 103578) and Dispase II (2.4 U ml-1, Roche 295825) supplemented with 2.5 mM CaCl2 for 60 min at 37ºC with periodic trituration. Monodispersed cells were resuspended in HBSS with 2% FBS (HBSS+ buffer), filtered through 30 µm FACS strainers (Miltenyi Biotech, Cat No: 130-041-407) and stained with appropriate antibodies and viability dyes (see Methods).<br>For E9.0 single-cell RNA-Seq data, Nkx2-1GFP-expressing domains (forebrain, thyroid, and lung tissues containing both GFP+ and GFP- lineages) were isolated from E9.0 embryos and monodispersed using the collagenase-dispase method as described above. Cell suspensions were filtered through 30 µm FACS strainers (Miltenyi Biotech, Cat No: 130-041-407) and stained with Calcein Blue (1:500 dilution) directly before sorting viable cells.<br>For in vitro experiments using the Nkx2-1-mCherry mouse embryonic stem cell line, Matrigel was disrupted on D14 using 2 mg ml-1 dispase for 1 h, then spun down at 100×g for 1 minute to enrich for clusters of epithelial cells. These cells were then broken down into a single-cell suspension using 0.25% trypsin and were stained with appropriate antibodies and viability dye (see Methods).
Instrument	Sorting was performed using the MoFlo (Astrios or Cytomation) and FACSARIA II SORP high speed cell sorters.

Software	Flow cytometry plots were generated using the FlowJo software (V10, Becton, Dickinson & Company).
Cell population abundance	For all sorts (in vivo and in vitro cells), purity tests were performed. For in vitro sorts, purity was generally higher than 95%. For E9.0 in vivo sorts, purity test were performed on the E9.0 forebrain Nkx2-1-GFP+ fraction and it was generally higher than 90%.
Gating strategy	The gating strategy is described in the Methods section and shown in the "Supplementary Information" document.

☒ Tick this box to confirm that a figure exemplifying the gating strategy is provided in the Supplementary Information.
