# Supplementary material for: The in vivo genetic program of murine primordial lung epithelial progenitors
Source: Nat Commun. 2020 Jan 31;11:635. doi: 10.1038/s41467-020-14348-3 (PMC6994558; doi:10.1038/s41467-020-14348-3)
Supplement: Supplementary file 4 — Supplementary Information [file 41467_2020_14348_MOESM4_ESM.pdf]

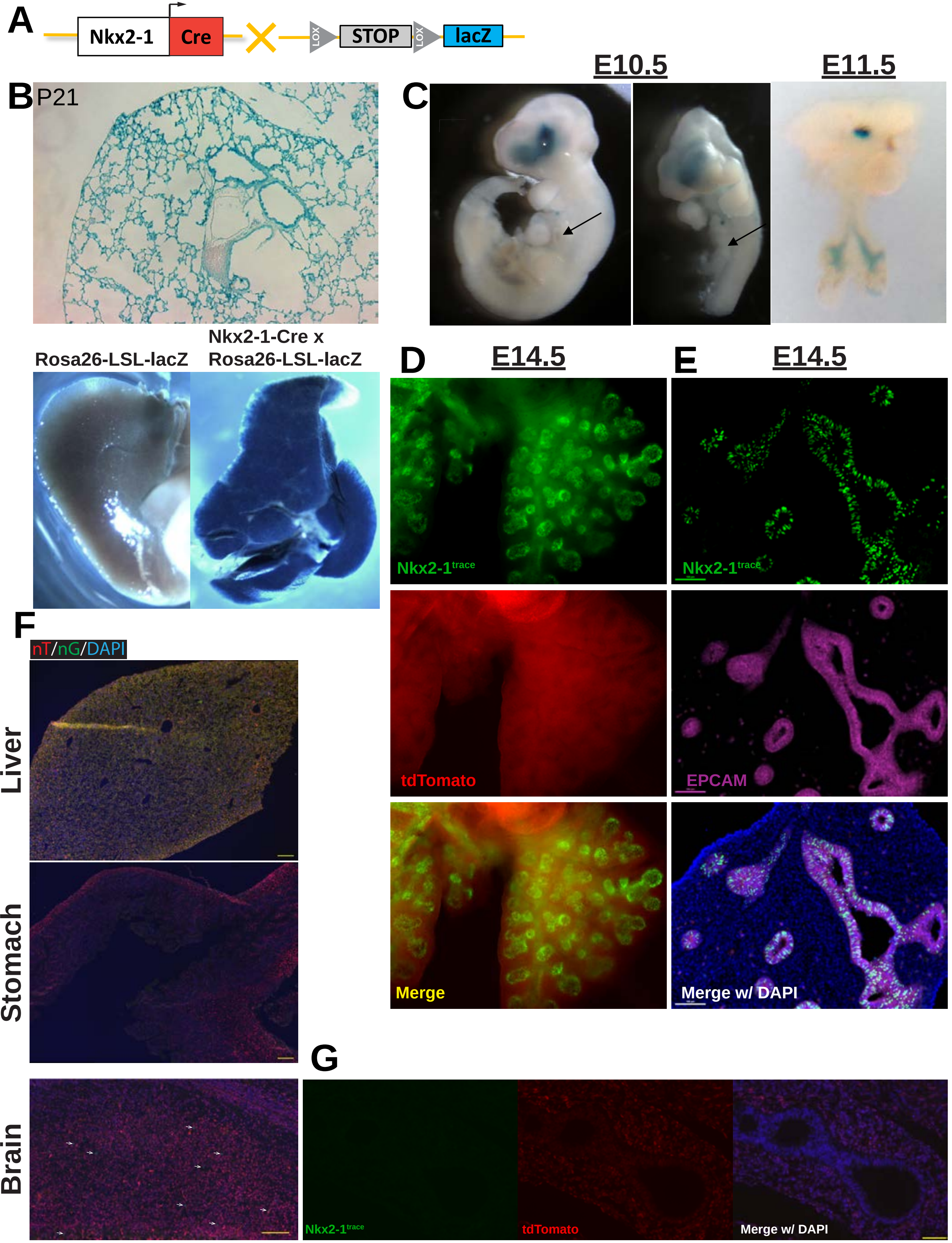

A
Nkx2-1
Cre
LOX
STOP
LOX
lacZ
B
P21
Rosa26-LSL-lacZ
Nkx2-1-Cre x
Rosa26-LSL-lacZ
C
E10.5
E11.5
D
E14.5
Nkx2-1trace
tdTomato
Merge
E
E14.5
Nkx2-1trace
EPCAM
Merge w/ DAPI
F
nT/nG/DAPI
Liver
Stomach
Brain
G
Nkx2-1trace
tdTomato
Merge w/ DAPI

Supplementary Figure 1. Nkx2-1 lineage trace marks exclusively epithelial cells within the developing mouse lung (A) Schematic of Nkx2-1-Cre; R26R mice. (B) Postnatal day 21 lungs from Nkx2-1-Cre; R26R mice show ubiquitous epithelial LacZ expression following staining with X-Gal. (C) Dark field micrographs show LacZ expression in forebrain and thyroid domains but not in the lung domain at E10.5 (left panel) whereas both thyroid and lung are LacZ-positive by E11.5 (right panel). (D) Nkx2-1^{trace} mice received two tamoxifen injections (E7.5 and E8.0) and sacrificed at E14.5. Epifluorescence micrographs of whole mount lineage-traced lungs at E14.5. Nkx2-1^{trace} (GFP^{+}) cells are cells that expressed Nkx2-1 at the moment of lung specification. Labeled cells appear along developing airways and distal branching tips. (E) Epifluorescence micrographs of E14.5 lung sections show Nkx2-1 lineage-labeled cells are exclusively epithelial cells. (F) Following tamoxifen administration at E8.75, there is absence of GFP^{+} cells in epifluorescent micrographs of E18.5 stomach and liver whereas GFP^{+} cells can be seen in E18.5 brain, Scale bars: 100 µm. Representative images from N= 3 embryos (G) Absence of GFP^{+} cells in epifluorescent micrographs of E13.5 lung labeled at E7.5, Scale bar: 50 µm. Representative image from N= 3 embryos.

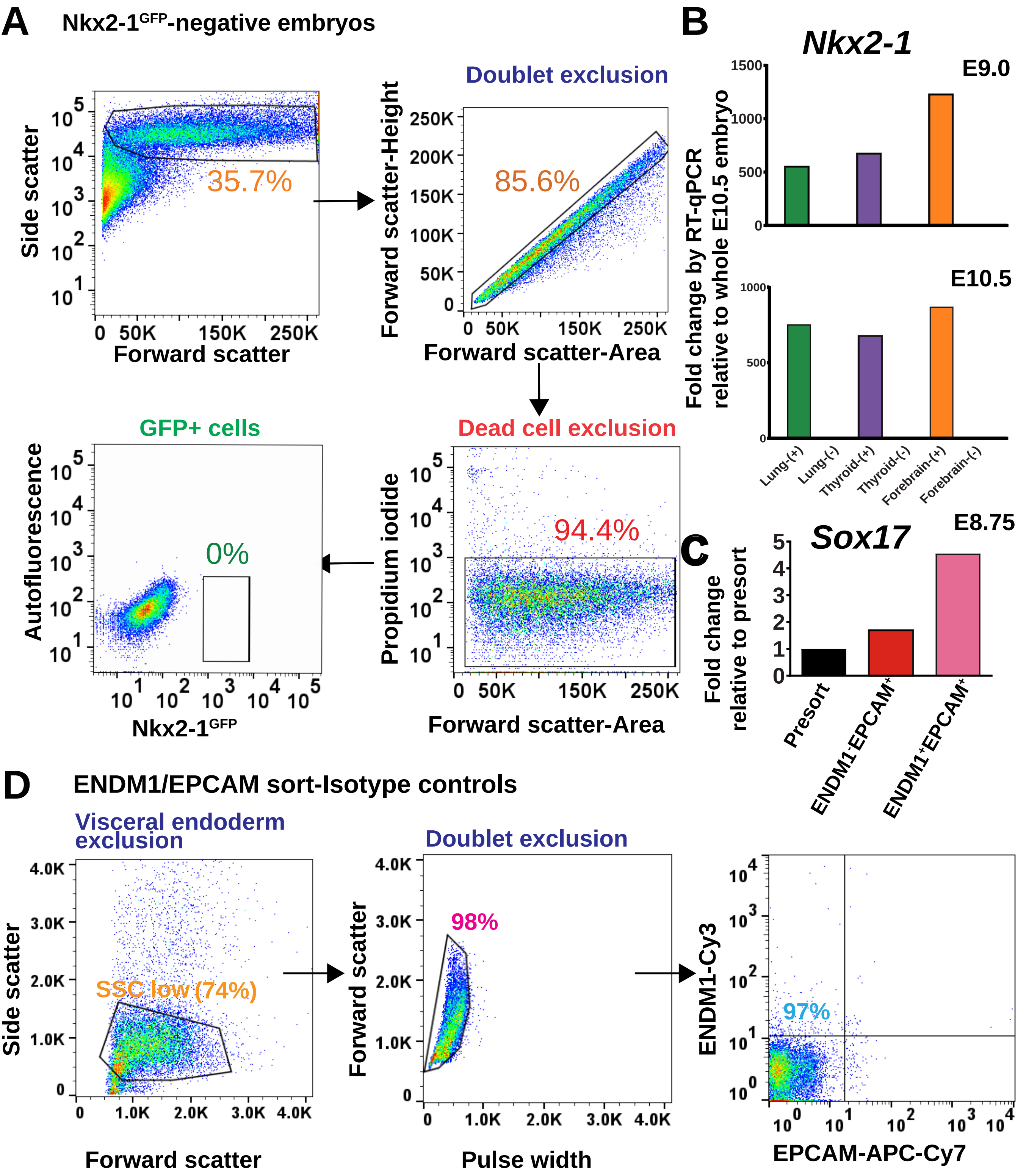

Supplementary Figure 2. Flow cytometry algorithms for the sorting to purity of cell populations used in population RNA-Seq analysis (A) Sorting algorithm for NKX2-1^{GFP+} and NKX2-1^{GFP-} cells for the RNA-Seq analysis in Figure 2. (B) RT-qPCR analysis of Nkx2-1 expression in sorted NKX2-1^{GFP+} and NKX2-1^{GFP-} fractions from the forebrain, thyroid and lung domains at E9.0 and E10.5. (C) RT-qPCR analysis of *Sox17* expression in the sorted foregut endoderm fraction. (D) Sorting algorithm for E8.25 foregut endoderm and ectoderm. The SSC^{low} gate is used to exclude visceral endoderm that also expresses ENDM1.

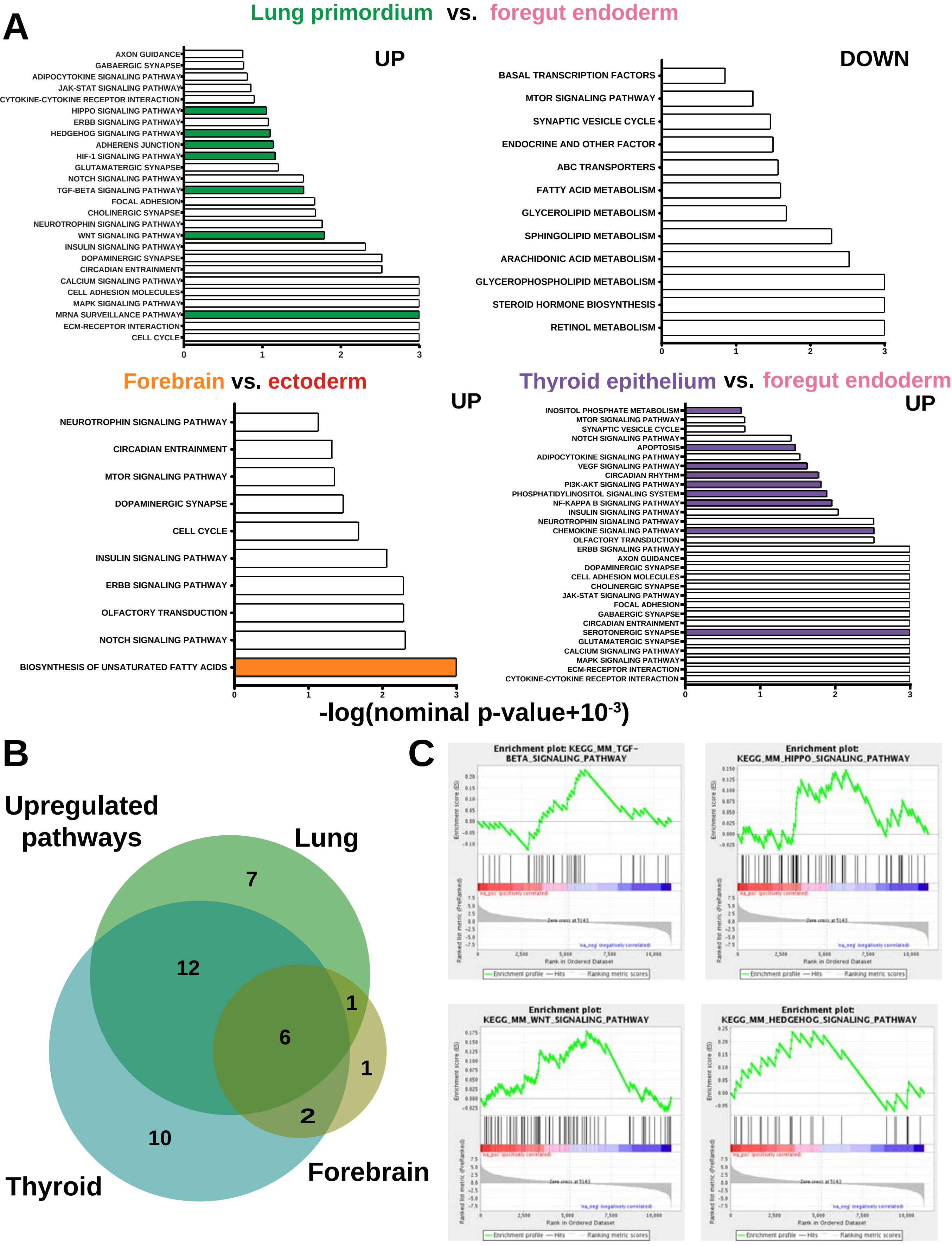
A
Lung primordium vs. foregut endoderm
UP
AXON GUIDANCE
GABAERGIC SYNAPSE
ADIPOCYTOKINE SIGNALING PATHWAY
JAK-STAT SIGNALING PATHWAY
CYTOKINE-CYTOKINE RECEPTOR INTERACTION
HIPPO SIGNALING PATHWAY
ERBB SIGNALING PATHWAY
HEDGEHOG SIGNALING PATHWAY
ADHERENS JUNCTION
HIF-1 SIGNALING PATHWAY
GLUTAMATERGIC SYNAPSE
NOTCH SIGNALING PATHWAY
TGF-BETA SIGNALING PATHWAY
FOCAL ADHESION
CHOLINERGIC SYNAPSE
NEUROTROPHIN SIGNALING PATHWAY
WNT SIGNALING PATHWAY
INSULIN SIGNALING PATHWAY
DOPAMINERGIC SYNAPSE
CIRCADIAN ENTRAINMENT
CALCIUM SIGNALING PATHWAY
CELL ADHESION MOLECULES
MAPK SIGNALING PATHWAY
MRNA SURVEILLANCE PATHWAY
ECM-RECEPTOR INTERACTION
CELL CYCLE
0
1
2
3
DOWN
BASAL TRANSCRIPTION FACTORS
MTOR SIGNALING PATHWAY
SYNAPTIC VESICLE CYCLE
ENDOCRINE AND OTHER FACTOR
ABC TRANSPORTERS
FATTY ACID METABOLISM
GLYCEROLIPID METABOLISM
SPHINGOLIPID METABOLISM
ARACHIDONIC ACID METABOLISM
GLYCEROPHOSPHOLIPID METABOLISM
STEROID HORMONE BIOSYNTHESIS
RETINOL METABOLISM
0
1
2
3
Forebrain vs. ectoderm
UP
NEUROTROPHIN SIGNALING PATHWAY
CIRCADIAN ENTRAINMENT
MTOR SIGNALING PATHWAY
DOPAMINERGIC SYNAPSE
CELL CYCLE
INSULIN SIGNALING PATHWAY
ERBB SIGNALING PATHWAY
OLFACTORY TRANSDUCTION
NOTCH SIGNALING PATHWAY
BIOSYNTHESIS OF UNSATURATED FATTY ACIDS
0
1
2
3
Thyroid epithelium vs. foregut endoderm
UP
INOSITOL PHOSPHATE METABOLISM
MTOR SIGNALING PATHWAY
SYNAPTIC VESICLE CYCLE
NOTCH SIGNALING PATHWAY
APOPTOSIS
ADIPOCYTOKINE SIGNALING PATHWAY
VEGF SIGNALING PATHWAY
CIRCADIAN RHYTHM
PI3K-AKT SIGNALING PATHWAY
PHOSPHATIDYLINOSITOL SIGNALING SYSTEM
NF-KAPPA B SIGNALING PATHWAY
INSULIN SIGNALING PATHWAY
NEUROTROPHIN SIGNALING PATHWAY
CHEMOKINE SIGNALING PATHWAY
OLFACTORY TRANSDUCTION
ERBB SIGNALING PATHWAY
AXON GUIDANCE
DOPAMINERGIC SYNAPSE
CELL ADHESION MOLECULES
CHOLINERGIC SYNAPSE
JAK-STAT SIGNALING PATHWAY
FOCAL ADHESION
GABAERGIC SYNAPSE
CIRCADIAN ENTRAINMENT
SEROTONERGIC SYNAPSE
GLUTAMATERGIC SYNAPSE
CALCIUM SIGNALING PATHWAY
MAPK SIGNALING PATHWAY
ECM-RECEPTOR INTERACTION
CYTOKINE-CYTOKINE RECEPTOR INTERACTION
0
1
2
3
-log(nominal p-value+10-3)
B
Upregulated pathways
Lung
7
12
1
6
1
2
10
Thyroid
Forebrain
C
Enrichment plot: KEGG_MM_TGF-BETA_SIGNALING_PATHWAY
Enrichment plot: KEGG_MM_HIPPO_SIGNALING_PATHWAY
Enrichment plot: KEGG_MM_WNT_SIGNALING_PATHWAY
Enrichment plot: KEGG_MM_HEDGEHOG_SIGNALING_PATHWAY

Supplementary Figure 3. GSEA of differentially regulated pathways in Nkx2-1+ embryonic progenitors (A) Identification of pathways that are differentially regulated in specified Nkx2-1+ embryonic populations by gene set enrichment analysis (GSEA). An FDR cutoff of 0.25 was used. A –log[p-value+10^{-3}] of 3 corresponds to nominal p-values of 0. Pathways specific to each population are highlighted with the respective color. (B) Venn diagram showing the number of pathways upregulated in each Nkx2-1^{+} progenitor population relative to their respective precursor stage (foregut endoderm or ectoderm). The pathways used to create the diagram are the ones shown in Figure 4A (FDR cutoff=0.25). (C) Gene enrichment plots for the pathways shown in the heat maps of Figure 4B.

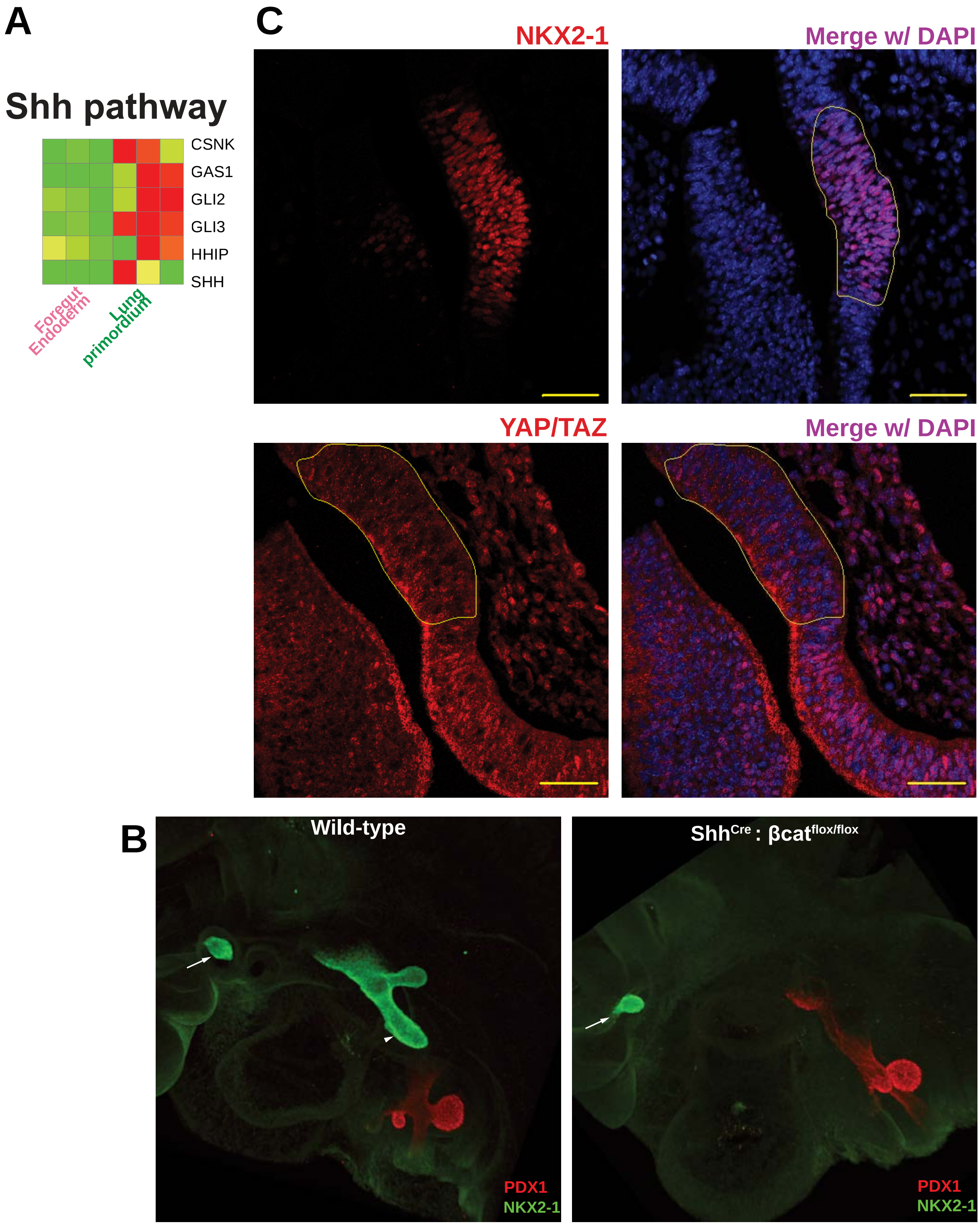

Supplementary Figure 4. Signaling pathways in lung specification (A) Heat map of selected transcripts for the Shh pathway. (B) Whole-mount confocal micrographs of uncultured, freshly isolated embryos at 25-28 somite stage. NKX2-1 expression demarcates the lung and thyroid domains and PDX1 expression demarcates the pancreatic domain; Arrow = Thyroid, Arrowhead = Lung. (C) Confocal micrographs of E9.0 mouse paraffin sections. Consecutive sections were stained for NKX2-1 and YAP/TAZ and the lung domain is demarcated by a yellow line.

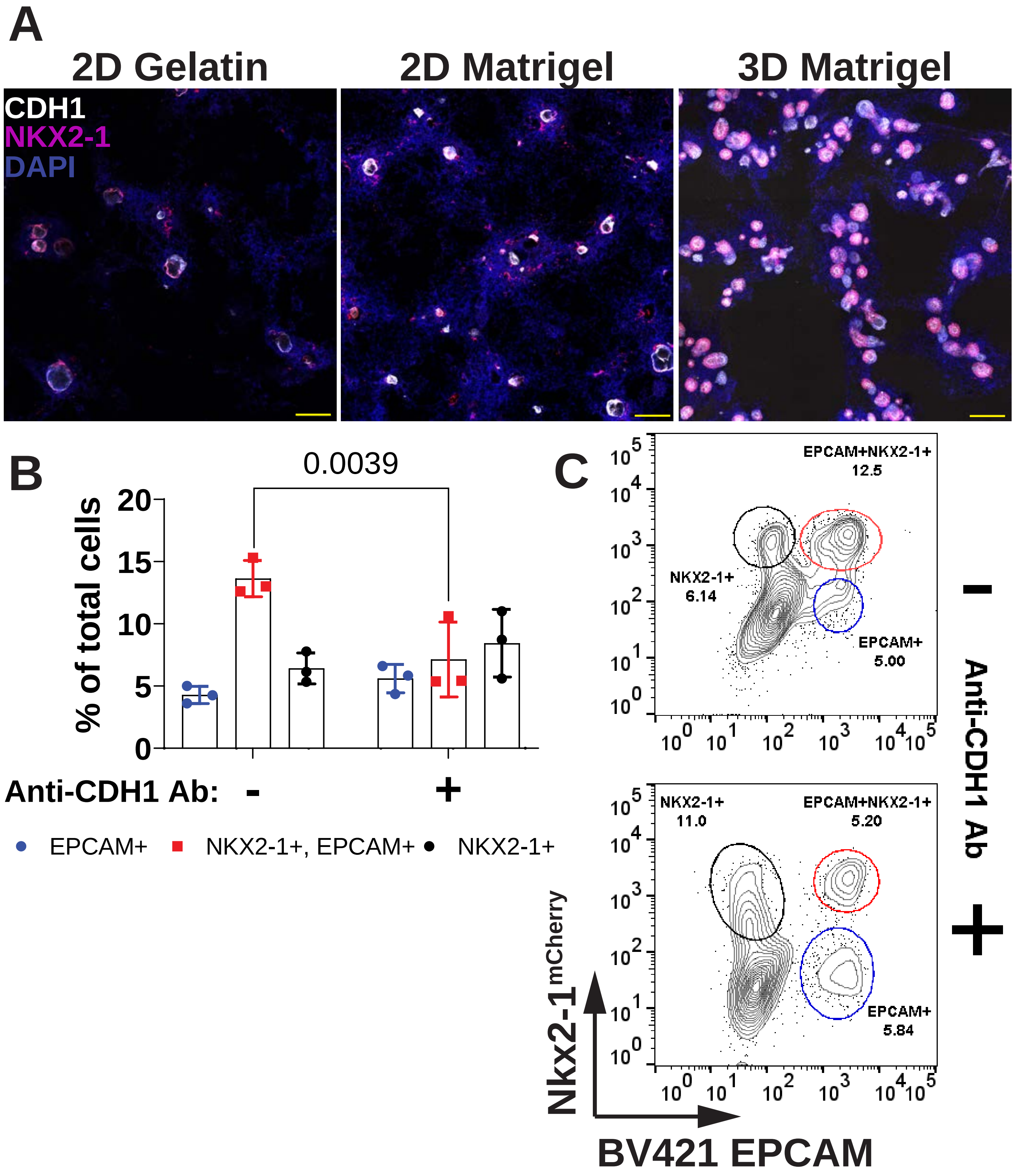

Supplementary Figure 5. Inhibition of adherens junctions leads to reduced lung progenitor derivation (A) Confocal micrographs of CDH1 (E-cadherin) and NKX2-1 staining in D14 cultures in lung specification media under three different substratum conditions, white staining indicates clusters of $CDH1^{+}$ cells, Scale bars: 200 μm. Representaitve images from N=3 independent experiments (B) Percentage of various D14 $NKX2\text{-}1^{+}$ and EPCAM+ populations on 3D Matrigel with or without antibody-mediated E-cadherin inhibi-tion from D6 to D14, p-value was calculated by 2-way ANOVA, N= 3 independent experiments, error bars represent standard deviations. C) Representative bivariate contour flow cytometry plots depicting the populations analyzed in (B)

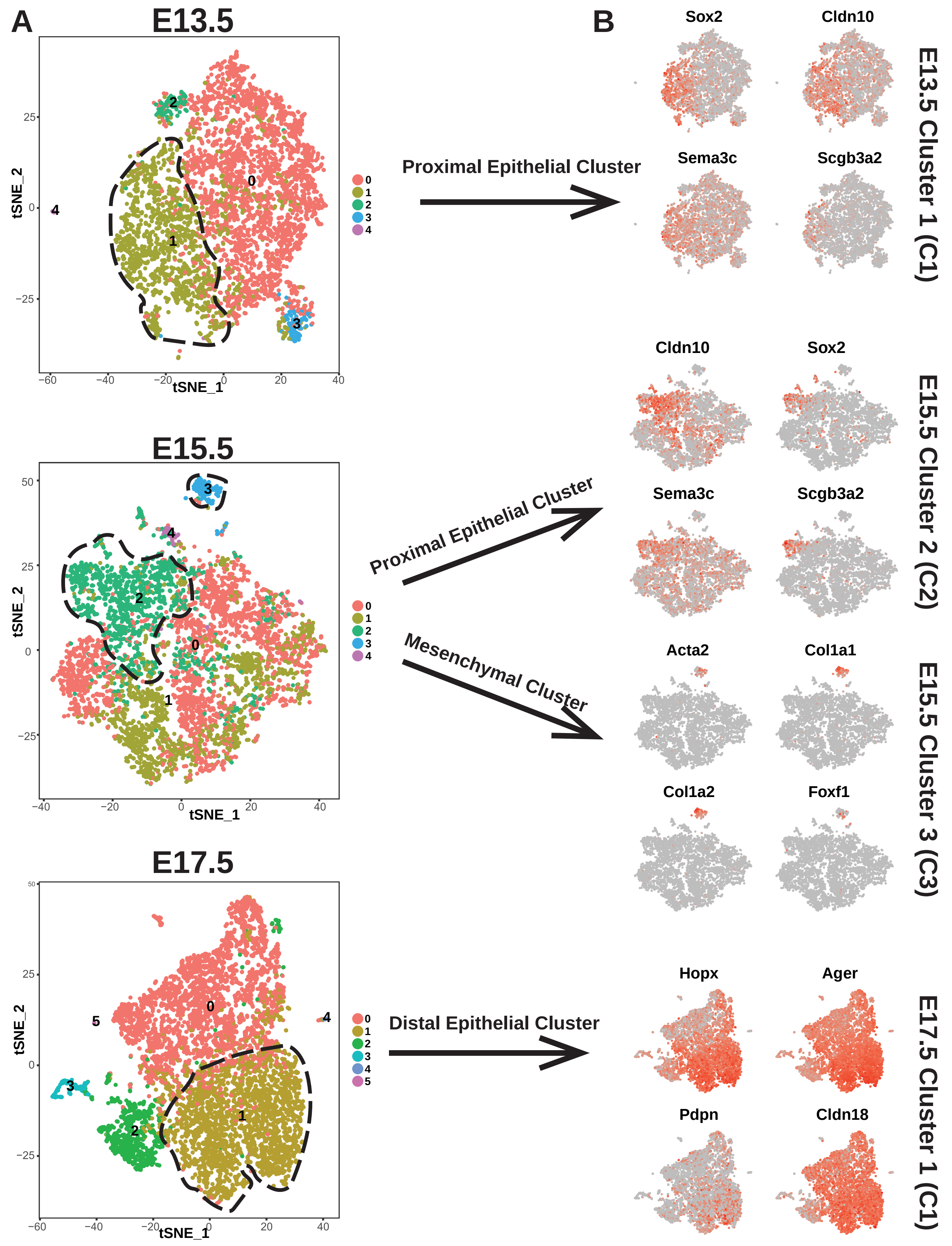

Supplementary Figure 6. Identification of cell clusters in single-cell RNA-Seq of embryonic lung at three time points (A) t-SNE plots at 0.5 resolution of single-cell RNA-Seq for highly enriched Nkx2-1^{+} cells from E13.5, E15.5 and E17.5 mouse lungs. (B) Gene expression of specific proximal or distal epithelial or mesenchymal markers of clusters identified in (A) and used for similarity analysis in Figure 6.

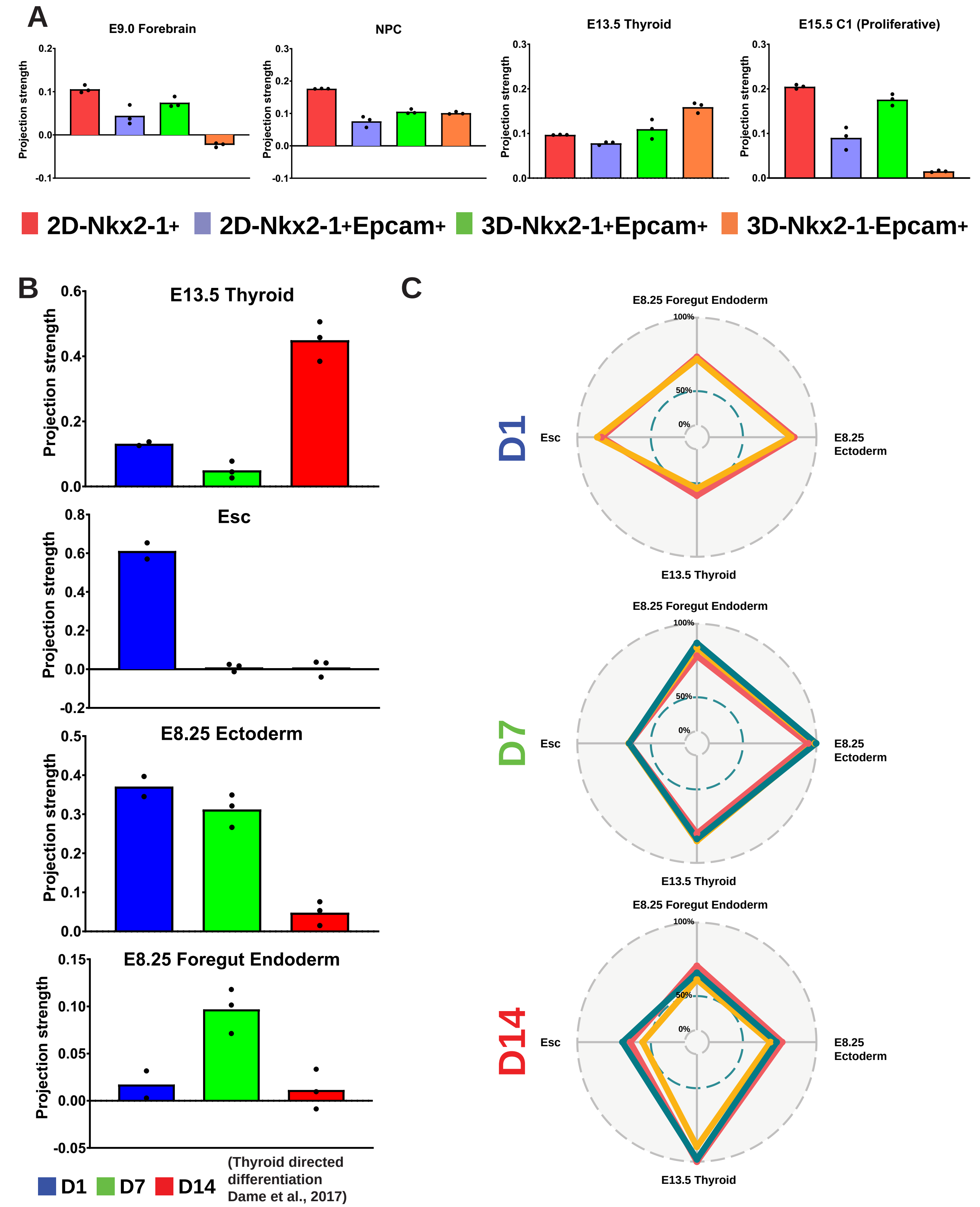

Supplementary Figure 7. Similarity analysis of control cell populations (A) Projection strength scores from LAP analysis for E9.0 Forebrain, neural progenitor cells (NPC), E13.5 thyroid, as well as an E15.5 lung proliferative cluster. The latter was characterized by enriched expression of genes such as *Ube2c, Cdk1, Cdc20, CCnb1, Cks2* and *Cenpf*. (B) LAP analysis using the current reference basis for the D1, D7 and D14 populations reported in Dame et al. [1]. Projections strength scores are similar to the ones calculated with the older reference basis. (C) Radar plots for gene set analysis projection scores show that this method reproduces the similarity analysis based on LAP.

Supplementary Table 1. RT-qPCR TaqMan Probes

**Expression Target**	**Catalogue Number**
18S rRNA	4319413e
Abca3	Mm00550501_m1
Epcam	Mm00493214_m1
Nkx2-1	4441117 Assay ID: AJLJIJO
Pax8	Mm00440623_m1
Scgb1a1	Mm00442046_m1
Scgb3a2	Mm00504412_m1
Sftpa	Mm00499170_m1
Stfpb	Mm00455681_m1
Sftpc	Mm00488144_m1
Trp63	Mm00495788_m1

Supplementary Table 2. KEGG Pathways used in GSEA analysis

KEGG_MM_ABC_TRANSPORTERS
KEGG_MM_ADHERENS_JUNCTION
KEGG_MM_ADIPOCYTOKINE_SIGNALING_PATHWAY
KEGG_MM_ANTIGEN_PROCESSING_AND_PRESENTATION
KEGG_MM_APOPTOSIS
KEGG_MM_ARACHIDONIC_ACID_METABOLISM
KEGG_MM_AXON_GUIDANCE
KEGG_MM_BASAL_TRANSCRIPTION_FACTORS
KEGG_MM_BIOSYNTHESIS_OF_UNSATURATED_FATTY_ACIDS
KEGG_MM_CALCIUM_SIGNALING_PATHWAY
KEGG_MM_CELL_ADHESION_MOLECULES
KEGG_MM_CELL_CYCLE
KEGG_MM_CHEMOKINE_SIGNALING_PATHWAY
KEGG_MM_CHOLINERGIC_SYNAPSE
KEGG_MM_CIRCADIAN_ENTRAINMENT
KEGG_MM_CIRCADIAN_RHYTHM
KEGG_MM_CYTOKINE-CYTOKINE_RECEPTOR_INTERACTION
KEGG_MM_CYTOSOLIC_DNA-SENSING_PATHWAY
KEGG_MM_DOPAMINERGIC_SYNAPSE
KEGG_MM_DORSO-VENTRAL_AXIS_FORMATION
KEGG_MM_ECM-RECEPTOR_INTERACTION
KEGG_MM_ENDOCRINE_AND_OTHER_FACTOR
KEGG_MM_ERBB_SIGNALING_PATHWAY
KEGG_MM_FATTY_ACID_BIOSYNTHESIS
KEGG_MM_FATTY_ACID_ELONGATION
KEGG_MM_FATTY_ACID_METABOLISM
KEGG_MM_FOCAL_ADHESION
KEGG_MM_GABAERGIC_SYNAPSE
KEGG_MM_GAP_JUNCTION
KEGG_MM_GLUTAMATERGIC_SYNAPSE
KEGG_MM_GLYCEROLIPID_METABOLISM
KEGG_MM_GLYCEROPHOSPHOLIPID_METABOLISM
KEGG_MM_GLYCOSAMINOGLYCAN_BIOSYNTHESIS_CHONDROITIN_DERMATAN_SULFATE
KEGG_MM_GLYCOSAMINOGLYCAN_BIOSYNTHESIS_HEPARAN_SULFATE_HEPARIN
KEGG_MM_GLYCOSAMINOGLYCAN_BIOSYNTHESIS_KERATAN_SULFATE
KEGG_MM_GLYCOSAMINOGLYCAN_DEGRADATION
KEGG_MM_GLYCOSYLPHOSPHATIDYLINOSITOL
KEGG_MM_HEDGEHOG_SIGNALING_PATHWAY
KEGG_MM_HIF-1_SIGNALING_PATHWAY
KEGG_MM_HIPPO_SIGNALING_PATHWAY
KEGG_MM_INOSITOL_PHOSPHATE_METABOLISM
KEGG_MM_INSULIN_SIGNALING_PATHWAY

KEGG_MM_JAK-STAT_SIGNALING_PATHWAY
KEGG_MM_LINOLEIC_ACID_METABOLISM
KEGG_MM_LIPOIC_ACID_METABOLISM
KEGG_MM_MAPK_SIGNALING_PATHWAY
KEGG_MM_METABOLIC_PATHWAYS
KEGG_MM_MRNA_SURVEILLANCE_PATHWAY
KEGG_MM_MTOR_SIGNALING_PATHWAY
KEGG_MM_MUCIN_TYPE_O-GLYCAN_BIOSYNTHESIS
KEGG_MM_NEUROTROPHIN_SIGNALING_PATHWAY
KEGG_MM_NF-KAPPA_B_SIGNALING_PATHWAY
KEGG_MM_NOTCH_SIGNALING_PATHWAY
KEGG_MM_OLFACTORY_TRANSDUCTION
KEGG_MM_P53_SIGNALING_PATHWAY
KEGG_MM_PENTOSE_PHOSPHATE_PATHWAY
KEGG_MM_PHOSPHATIDYLINOSITOL_SIGNALING_SYSTEM
KEGG_MM_PI3K-AKT_SIGNALING_PATHWAY
KEGG_MM_REGULATION_OF_ACTIN_CYTOSKELETON
KEGG_MM_RETINOL_METABOLISM
KEGG_MM_SEROTONERGIC_SYNAPSE
KEGG_MM_SPHINGOLIPID_METABOLISM
KEGG_MM_STEROID_HORMONE_BIOSYNTHESIS
KEGG_MM_SYNAPTIC_VESICLE_CYCLE
KEGG_MM_TGF-BETA_SIGNALING_PATHWAY
KEGG_MM_TIGHT_JUNCTION
KEGG_MM_VEGF_SIGNALING_PATHWAY
KEGG_MM_VITAMIN_B6_METABOLISM
KEGG_MM_WNT_SIGNALING_PATHWAY

Supplementary References

1. Dame K, et al. Thyroid Progenitors Are Robustly Derived from Embryonic Stem Cells through Transient, Developmental Stage-Specific Overexpression of Nkx2-1. *Stem Cell Rep* 8, 216-225 (2017).
